# From Seeds to Fibrils and Back: Fragmentation as an Overlooked Step in the Propagation of Prions and Prion-Like Proteins

**DOI:** 10.3390/biom10091305

**Published:** 2020-09-10

**Authors:** Cristóbal Marrero-Winkens, Charu Sankaran, Hermann M. Schätzl

**Affiliations:** 1 Department of Comparative Biology & Experimental Medicine, Faculty of Veterinary Medicine, University of Calgary, Calgary, AB T2N 4Z6, Canada; cristobal.marrerowin@ucalgary.ca (C.M.-W.); saambavi.sankaran@ucalgary.ca (C.S.); 2 Calgary Prion Research Unit, University of Calgary, Calgary, AB T2N 4Z6, Canada; 3 Hotchkiss Brain Institute, University of Calgary, Calgary, AB T2N 4Z6, Canada; 4 Faculty of Science, University of British Columbia, Vancouver, BC V6T 1Z4, Canada

**Keywords:** neurodegeneration, protein misfolding, amyloid, fragmentation, disaggregation, prion diseases, Alzheimer’s disease, Parkinson’s disease, Hsp110, autophagy

## Abstract

Many devastating neurodegenerative diseases are driven by the misfolding of normal proteins into a pathogenic abnormal conformation. Examples of such protein misfolding diseases include Alzheimer’s disease, Parkinson’s disease, Huntington’s disease, amyotrophic lateral sclerosis, and prion diseases. The misfolded proteins involved in these diseases form self-templating oligomeric assemblies that recruit further correctly folded protein and induce their conversion. Over time, this leads to the formation of high molecular and mostly fibrillar aggregates that are increasingly inefficient at converting normal protein. Evidence from a multitude of in vitro models suggests that fibrils are fragmented to form new seeds, which can convert further normal protein and also spread to neighboring cells as observed in vivo. While fragmentation and seed generation were suggested as crucial steps in aggregate formation decades ago, the biological pathways involved remain largely unknown. Here, we show that mechanisms of aggregate clearance—namely the mammalian Hsp70–Hsp40–Hsp110 tri-chaperone system, macro-autophagy, and the proteasome system—may not only be protective, but also play a role in fragmentation. We further review the challenges that exist in determining the precise contribution of these mechanisms to protein misfolding diseases and suggest future directions to resolve these issues.

## 1. Introduction

Protein misfolding disorders (PMDs) are the most common form of neurodegenerative disease [1] and involve the improper folding of specific proteins, resulting in protein aggregation, toxicity, and neuronal loss [2]. Common PMDs include Alzheimer’s disease, Parkinson’s disease, Lewy-body dementia, polyglutamine diseases (e.g., Huntington’s disease), amyotrophic lateral sclerosis, and prion diseases such as Creutzfeldt–Jakob disease [1]. Taken together, these neurodegenerative diseases are highly prevalent and place a great financial and social toll on our aging society [3]. For example, there are approximately 5.3 million cases of Alzheimer’s disease in the US, and this figure is projected to rise greatly in the future. With an annual per patient cost of 45,805 to 61,525 USD, a total cost of 243 billion USD federally per year represents a conservative estimate [3]. Parkinson’s disease on the other hand has approximately 630,000–1,000,000 cases in the US, with a per patient cost of $13,786 resulting in a total cost of approximately 8.1 billion USD [3].

Prion diseases were amongst the first diseases for which a link to protein misfolding was established. As postulated by the protein-only hypothesis, prions replicate without the involvement of nucleic acids and are solely composed of a protein [4,5,6]. Prions arise from the conformational conversion of the cellular prion protein (PrP^C^) into a misfolded isoform known as PrP^Sc^. Crucially, PrP^Sc^ is self-templating, which means it can induce further native protein to adopt its misfolded conformation [4]. As has been reviewed elsewhere [7,8], this and many other features of prions appear to be shared by proteins driving other “prion-like” neurodegenerative PMDs. A brief overview of selected PMDs and the proteins involved may be found in Table 1 and in the glossary at the end of the present review.

Models based on kinetic data suggest that the conversion of native protein is catalyzed largely by oligomeric ‘seeds’, rather than fibrillar aggregates [9]. Consequently, the fragmentation of fibrils into seeds has been described as a crucial step during the propagation of prions and prion-like misfolded proteins. Additionally, oligomeric species are also considered to be the main mediators of neurotoxicity in PMDs. While the subject of toxicity is beyond the scope of this review, we refer the interested reader to [10,11,12,13] for a detailed discussion of this topic.

In the present review, we aim to summarize the experimental evidence supporting fragmentation as a critical step in the propagation of prions and prion-like misfolded proteins. This has largely originated from the development of in vitro amplification techniques for misfolded proteins, as well as the study of yeast prions. We further aim to summarize the field’s current understanding of the cellular pathways that might facilitate fragmentation, placing particular emphasis on chaperone systems and macro-autophagy.

## 2. Evidence for Fragmentation in the Propagation of Disease-Associated Protein Aggregates

**Figure 1:**
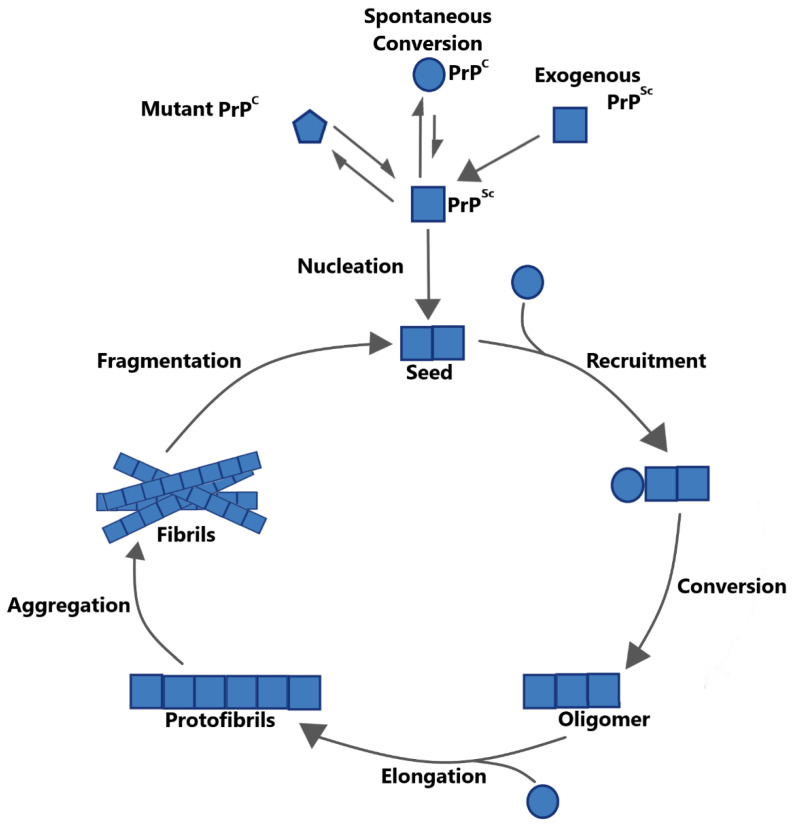
Seeded nucleation model exemplified by the cellular prion protein and its misfolded isoform (PrP^C^/PrP^Sc^). The formation of PrP^Sc^, and thus prion disease, can be initiated by the spontaneous conversion of wild-type PrP^C^ (circles) by inherited mutations in the PRNP gene (pentagon) or by infection with exogenous PrP^Sc^ (squares). Initial PrP^Sc^ undergoes nucleation forming a seed, which then recruits and converts further PrP^C^. This leads to the formation of oligomers, protofibrils, and ultimately, fibrillar aggregates. Due to the low ratio of exposed ends to their mass, fibrils are not efficient at converting further PrP^C^. Thus, fragmentation is required to generate new seeds, allowing the cycle to restart.

Kinetic models have long suggested that the propagation of misfolded proteins and the formation of amyloids might occur via seeded nucleation (Figure 1) [9,14,15]. The seeded nucleation model proposes that correctly folded and misfolded isoforms of pathogenic proteins are in equilibrium, with the correctly folded isoform normally being dominant. The slow and rate-limiting step in the development of disease lies in the formation of small aggregates of misfolded protein. These aggregates, termed nuclei, recruit and convert correctly folded protein at their ends, eventually forming larger fibrillar aggregates. Due to the low number of exposed ends relative to their mass, such fibrils are comparatively inefficient at recruiting and converting correctly folded protein. For the conversion process to continue, fibrils must give rise to more nuclei, or ‘seeds’, most likely by way of fragmentation. As envisioned by Jarret and Lansbury (1993) [9], in vitro amplification techniques for various amyloidogenic proteins have confirmed central elements of the seeded nucleation model. Further evidence for this model has come from the investigation of yeast prions and their interactions with chaperones. In this section, we attempt to summarize these two lines of evidence, while focusing particularly on the role played by fragmentation processes.

### 2.1. Evidence from In Vitro Amplification Techniques

Techniques allowing in vitro amplification of misfolded proteins are of great interest to the broader field of protein misfolding disorders (PMDs). Among their many applications, such techniques provide a diagnostic tool for the detection of misfolded proteins [16,17], as well as an experimental model for their propagation. As the latter, in vitro amplification techniques can be applied to the investigation of basic prion biology or to the study of potential therapies [18].

In vitro amplification is most established for PrP^Sc^, where it is used clinically in conjunction with other diagnostic methods [19]. Two major techniques exist, which differ in the substrate for conversion and in the read-out used: protein misfolding cyclic amplification (PMCA) and real-time quaking-induced conversion (RT-QuIC). PMCA, first developed by Soto and colleagues, involves incubating healthy brain homogenate or PrP^C^ extracted from healthy brain with highly diluted prion-infected brain [20]. PMCA results in the formation of infectious proteinase K (PK)-resistant PrP (PrP^res^), which has to be detected by immunoblot in a manner analogous to PrP^Sc^ [21,22]. PMCA is commonly carried out in several rounds. Combined with the need for immunoblot, this makes it a time-consuming assay. Therefore, significant efforts were made to increase the practicality of in vitro prion conversion assays. Based on the very first description of in vitro conversion [23], Caughey and colleagues developed the RT-QuIC assay [24,25,26,27].

During RT-QuIC, recombinant PrP (rPrP) is used a substrate for conversion, and Thioflavin T (ThT) fluorescence is measured in real time as a read-out for the formation of β-sheeted aggregates.

Interestingly, both assays require the intermittent application of mechanical forces. These are assumed to cause the breakage of aggregates in line with the seeded nucleation model proposed by Jarret and Lansbury (1993) [9]. For PMCA, shearing force is provided by sonication, while RT-QuIC employs shaking. With regard to PMCA, Saborio et al. (2001) [20] note that no formation of PrP^res^ is detectable if the PMCA mixture is not subjected to sonication. Similarly, no detectable conversion was reported for a precursor of the current RT-QuIC protocol when shaking was omitted [25]. Notably, both PMCA and RT-QuIC are highly efficient and require only minute quantities of PrP^Sc^ relative to PrP^C^ [26,28]. This is in stark contrast with previous techniques, which require a large excess of PrP^Sc^ and did not include a mechanical breakage of aggregates [23].

Since their inception, PMCA and RT-QuIC have also been adapted for the detection and amplification of other misfolded proteins. For amyloid-β (Aβ)—a major constituent of plaques found in patients with Alzheimer’s diseases (AD)—Soto and colleagues have developed an RT-QuIC-like assay, which employs recombinant Aβ_1-42_ as a substrate, ThT fluorescence as a read-out, and intermittent shaking to fragment aggregates [29]. Both synthetic pre-formed fibrils and samples of human cerebrospinal fluid (CSF) were successfully used as seeds for conversion.

Detecting the main component of neurofibrillary tangles (NFTs) associated with AD or various tauopathies—i.e., misfolded tau—is hindered by the presence of multiple tau isoforms arising from alternative splicing [30] and by the differential involvement of those isoforms in different neurodegenerative diseases. Nonetheless, several in vitro amplification assays for tau have been reported [31,32,33,34]. In the first reported RT-QuIC-like assay for tau, Meyer et al. (2014) [31] were able to detect the aggregation of synthetic monomeric tau using either synthetic tau fibrils or homogenized human AD brain as a seed. In line with kinetic data suggesting fibril fragmentation as the main pathway for the propagation of tau aggregates [35], the authors note that including intervals of sonication greatly improved the assay’s sensitivity. Specifically, 40 rounds of 5s sonication pulses increased the detection limit for synthetic K18 tau seeds from 0.1% to 0.00001% of monomer equivalent. Further refinements made by others have enabled researchers to not only detect misfolded tau, but to also distinguish between different tauopathies based on the different kinds of tau aggregates involved [32,33,34].

In vitro amplification has also been performed successfully for α-syn [36,37,38,39], which is the main component of inclusions found in patients with Parkinson’s disease (PD), Lewy body dementia (LBD), or multiple systems atrophy (MSA). In 2016, Green and colleagues [36] first reported an RT-QuIC-like assay capable of detecting misfolded α-syn in the CSF of patients with PD or LBD. A modified more rapid protocol with similar sensitivity and specificity was described by Groveman et al. (2018) [38], and more recently an in vitro assay that is able to distinguish PD patients from MSA patients has been reported [39].

The adaptability of PMCA and RT-QuIC to other PMDs supports the notion of seeded nucleation as a shared mechanism in the propagation of proteins associated with PMDs. Most notably, all assays intermittently apply mechanical forces to the reaction mixture to achieve efficient amplification. A handful of studies have directly compared protocols with and without mechanical forces. These studies have consistently found a reduced efficiency of conversion in the absence of intermittent mechanical forces [20,25,31]. Taken together, the development of the in vitro amplification techniques described above provides evidence for fragmentation as an essential step in the propagation of amyloidogenic misfolded proteins.

### 2.2. Evidence from Propagation of Prions in Yeast

The biology of yeast prions has recently been reviewed elsewhere [40,41,42,43]. Hence, we will limit our discussion to the role of fragmentation in yeast prion propagation.

The first described and most studied yeast prion phenotypes are [*psi*^+^] and [*URE3*], which arise from conformational changes in the proteins Sup35p and Ure2p, respectively [44]. The heat shock protein 104 (Hsp104) chaperone has been identified as essential for the propagation of [*psi*^+^], as the knockout of Hsp104 causes a loss of [*psi*^+^] over several cell divisions [45]. In cells lacking Hsp104, the phenotype could also not be induced by the overexpression of *SUP35*, which had previously been reported to do so in wild-type (WT) cells [46]. Similar experiments involving [*URE3*] also found an absolute requirement for Hsp104 to maintain the prion phenotype [47].

The fragmentation of misfolded Sup35p aggregates was suggested as the mechanism of action for Hsp104 based on the observation that the proportion of soluble Sup35p is increased in lysates of cells overexpressing Hsp104 [48]. Seminal work by Lindquist and colleagues later confirmed that Hsp104 does catalyze the formation of Sup35p seeds [49]. More broadly, the group also identified Hsp104 as the main player of a yeast machinery that disassembles stress-induced protein aggregates of all kinds [50].

Interestingly, high levels of Hsp104 overexpression were also reported to cure [*psi^+^*] [45]. This was initially attributed to a complete solubilization of aggregates [45,50], but no such curing effect of Hsp104 overexpression was observed for other yeast prions, and the mechanism behind the unusual dose relationship between [*psi^+^*] and Hsp104 is still a matter of debate [41,47].

Overall, the evidence outlined above strongly indicates that yeast prions rely on fragmentation by chaperones for their propagation. The extent to which this translates to the propagation of mammalian prions in vivo cannot be determined with certainty. Since Hsp104 does not have a mammalian homologue, alternative cellular pathways or machineries would have to fulfill its function in mammalian cells. Interestingly, the yeast Sup35 prion domain can also propagate as a prion in mammalian cells, suggesting that cellular mechanisms support prion-like inheritance in the mammalian cytosol [51]. Given the extensive similarities between yeast prions and mammalian prions, we believe that the wider principle of propagation by fragmentation and seed generation applies to mammalian prions and prion-like proteins as well.

### 2.3. Secondary Nucleation as an Alternative Mode of Propagation

While the evidence outlined above supports fragmentation as a mode of propagation relevant to all misfolded proteins, it should be noted that secondary nucleation may be predominant for certain proteins under certain conditions [52,53,54]. Secondary nucleation differs from fragmentation in that new seeds are generated by the recruitment of monomers to the surface of an existing aggregate where they subsequently undergo conformational changes and nucleation. A detailed analysis of the relative contributions of fragmentation and secondary nucleation is beyond the scope of this review, but we refer the interested reader to [55] where this has recently been discussed.

## 3. On the Biological Pathways That May Be Involved in the Fragmentation of Mammalian Prions

**Figure 2:**
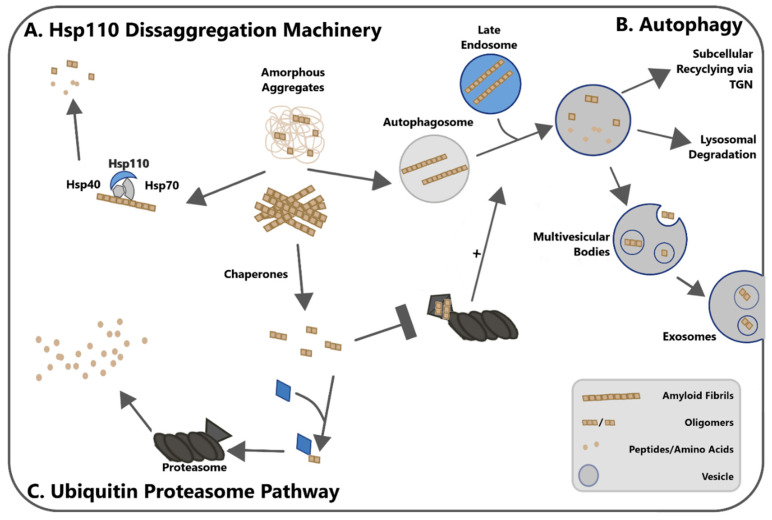
Schematic representation of cellular mechanisms involved in protein degradation and their proposed roles in fragmentation and seed generation. All pathways may start with either fibrillar aggregates or amorphous aggregates containing seeding-competent oligomers. (**A**) The Hsp70–Hsp40–Hsp110 disaggregase. The tri-chaperone complex binds to aggregates and degrades the fibrils into smaller units through its disaggregase activity. Additionally, the disaggregation of amorphous aggregates may liberate seeding-competent oligomers. (**B**) The autophagy–lysosomal pathway. Some aggregation-prone are found in the cytosol, while others are located in endosomal vesicles. Autophagosomes fuse with late endosomes, and substrates can then undergo several fates: lysosomal degradation, exosomal release from the cell, or recycling to other cellular compartments. (**C**) The ubiquitin–proteasome system (UPS). The proteasome degrades polyubiquitinated oligomers or monomers of misfolded proteins. Given this substrate range, a direct involvement in fragmentation is unlikely, but oligomeric species of certain misfolded proteins have been reported to inhibit the UPS. Recent research has suggested that there may be ‘cross-talk’ between the UPS and autophagy, whereby inhibition of the UPS stimulates autophagic flux.

Cells regulate the synthesis, folding, conformation, and degradation of proteins in order to maintain proteostasis, i.e., protein homeostasis [56]. With respect to amyloidogenic proteins, the cellular pathways involved in protein degradation may aid the formation of seeding-competent intermediates from fibrils (Figure 2). Due to this possible dual role, these pathways have also been suggested to mediate fragmentation, which is necessary for the propagation of misfolded proteins. In the present section, we attempt to summarize the experimental evidence underlying this view, while focusing specifically on the Hsp70–Hsp40–Hsp110 tri-chaperone system and macro-autophagy.

It should be noted that these cellular mechanisms may provide seeds—and thus aid protein conversion—not only by fragmenting amyloid fibrils, but also by liberating seeding-competent oligomers from amorphous aggregates or from non-fibrillar structured aggregates. In the present section, we refer mainly to fibrils, but the concepts may extend analogously to such non-fibrillar aggregates.

### 3.1. The Hsp70–Hsp40–Hsp110 Tri-Chaperone System—A Mammalian Disaggregase with Conflicting Roles?

#### 3.1.1. Mechanistic INSIGHTS from In Vitro Studies

As outlined above, yeast possess a potent disaggregase machinery in the form of Hsp104, which collaborates with Hsp70 and Hsp40 to rescue aggregated proteins. For instance, homologues of Hsp104 are found in bacteria and plants but not in mammals [57]. It had long been hypothesized that a functionally equivalent system should exist in mammals, but it was not until 2011 that a chaperone-based mammalian disaggregase was described. Shorter (2011) [58] showed that cytosolic preparations of mammalian cells could re-activate urea-denatured firefly luciferase and heat-denatured green fluorescent protein (GFP). Crucially, this activity was lost upon the immunodepletion of Hsc70 (a constitutively expressed chaperone of the Hsp70 family), DNAJB1 (an Hsp40 family member), or Hsp110 family members Hsp105 or Apg-2. Interestingly, recombinant Hsp70 and Hsp40 together did not perform as well as the cytosol preparations in a pure re-activation assay. Instead, this required the addition of Hsp110 family members, which suggests that Hsp70, Hsp40, and Hsp110 together form a mammalian tri-chaperone system that acts as a disaggregase. This finding was echoed by Bukau and colleagues whose in vitro studies showed that this tri-chaperone system could also disaggregate thermally-denatured luciferase and malate dehydrogenase (MDH) [59]. The authors further reported that RNAi-mediated knockdown of Hsp110 blocked the dissolution of aggregates in heat-shocked *Caenorhabditis elegans* [59], thereby providing first in vivo evidence for a chaperone-based disaggregase in metazoa.

Subsequent work by Bukau and colleagues showed that the human tri-chaperone system can also fragment fibrillar aggregates [60,61] in vitro. Gao et al. (2015) [60] incubated pre-formed α-syn fibrils with physiogically attainable concentrations of a chaperone complement containing Hsc70, DNAJB1, the Hsp110 family member Apg-2, and an ATP-regeneration system. Then, the fraction of re-solubilized α-syn was determined by centrifugation, SDS-PAGE, and immunoblotting. The authors found that disaggregation and solubilization increased with both the incubation time and the concentration of chaperone complement added, reaching up to 80% after a 24 h incubation period. Equivalent experiments on α-syn fibrils isolated from *C. elegans* expressing α-syn tagged with YFP also found some α-syn disaggregation after a 4 h incubation period. Nachman et al. (2020) [61] treated various kinds of tau fibrils with the Hsc70/DNAJB1/Apg-2 chaperone complement and subsequently carried out ultracentrifugation. This separates mono- and oligomeric tau (found in the supernatant fraction) from remaining fibrillar tau (found in the pellet fraction). Following 20 h incubation of pre-formed tau fibrils with the chaperone complement, 40–50% of tau was found in the supernatant fraction, which demonstrates the efficient disassembly of fibrils. Remarkably, the incubation of chaperones with tau fibrils extracted from cortical gray matter of AD patients also resulted in a shift of approximately 60% of tau to the supernatant. Overall, these data suggest that the Hsp70–Hsp40–Hsp110 system cannot only act on unstructured amorphous aggregates, but also on highly ordered amyloids relevant to PMDs.

In their studies on α-syn fibrils, Gao et al. (2015) [60] also examined whether the Hsc70/DNAJB1/Apg-2 system depolymerized fibrils by removing monomers from fibril ends or fragmented fibrils by extracting monomers from within fibrils. Combining the results of three distinct approaches—negative-stain electron microscopy, sedimentation velocity-gradient centrifugation, and capping fibrils with biotinylated α-syn—the authors provided evidence for a dual mechanism that includes both depolymerization and fragmentation. Nachman et al. (2020) [61] used sequential centrifugation to examine the size distribution of tau aggregates arising from the chaperone-mediated disaggregation of pre-formed tau fibrils. The authors found re-solubilized tau to consist largely of monomers, dimers, and tetramers. Importantly, the supernatant of chaperone-treated tau fibrils could seed tau aggregation in P301S tau-expressing HEK293 biosensor cells, whereas the supernatant of untreated fibrils or fibrillar aggregates themselves could not. While it remains to be determined whether similar seeding-competent species are generated by the Hsp70–Hsp40–Hsp110 system in vivo, these observations put the tri-chaperone system on the map as a potential mediator of fragmentation and, thus, as a potential player in the propagation of misfolded proteins.

A recent study on Hsp110 modulation in *C. elegans* expressing α-syn tagged with yellow fluorescent protein (YFP) has provided further evidence for a role of Hsp110 and its co-chaperones in seed generation and fragmentation [62]. Specifically, Tittelmeier et al. (2020) [62] show that a short hairpin RNA (shRNA)-mediated knockdown of Hsp110 reduces the number of foci in *C. elegans* muscle cells and rescues the motility defects typically associated with such aggregates. Remarkably, the authors reported that cell-to-cell transfer of aggregates was only seen in 50–60% of animals after the knockdown of Hsp110.

Similarly, we have observed small interfering RNA (siRNA)-mediated knockdown of Hsp110 to reduce the levels of PrP^Sc^ in various stably prion-infected neuronal and non-neuronal cell lines within 96 h (Marrero-Winkens, C.; Sankaran, C. and Schätzl H.M, personal communication). Furthermore, we observed a reduced susceptibility to de novo prion infection with mouse-adapted scrapie prions in neuroblastoma (N2a) cells devoid of either Apg-2 or Hsp105. Conversely, the transient overexpression of Hsp110 dose-dependently increased the levels of PrP^Sc^ in N2a cells infected with mouse-adapted scrapie prions. Although preliminary, these data are reminiscent of the effects of Hsp104 modulation on yeast prions and would be consistent with a role of the tri-chaperone system in mammalian prion propagation.

Overall, the combined data from cell-free systems, cultured mammalian cells, and *C. elegans* suggest that Hsp70–Hsp40–Hsp110 may provide fragmentation activity for mammalian amyloidogenic proteins. Further studies should be conducted on the various aggregation-prone proteins in a variety of in vitro systems. This will not only reveal whether such fragmentation activity is truly universal, but it may additionally point to ways in which it can be modulated to slow or prevent the propagation of prions and prion-like proteins.

Mechanistically, it is known that Hsp70 undergoes repeated protein binding-and-release cycles due to conformational changes in its N-terminal substrate binding domain (SBD) following adenosine triphosphate (ATP)-binding, ATP-hydrolysis, and adenosine diphosphate (ADP)-release from its nucleotide binding domain (NBD) [63,64,65,66]. In the ATP-bound state, the affinity for substrate is low, and ATPase activity is also low. Interaction with an Hsp40 family member promotes ATP hydrolysis, which leads to substrate recruitment by causing the alpha-helical lid domain of Hsp70 to close over its beta-sandwich substrate-binding pocket. In the ADP-bound state, both substrate release and ADP release are slow, which causes trapping of the substrate. Interaction with a nucleotide exchange factor (NEF), such as Hsp110, promotes ADP release, allowing for release of the substrate and re-starting the cycle. Recent work by Bukau and colleagues has shown that while Hsp110 is crucial for effective disaggregation, it is the availability of Hsp40 family members that drives aggregate specificity [67]. While it remains largely unknown how Hsp70 mediates disaggregation through client binding-and-release cycles, an attractive ‘nucleation model’ has recently been proposed. Briefly, it proposes Hsp40 binding to the surface of an aggregate as the initial step, followed by the recruitment of Hsp70 and Hsp110. Crucially, the oligomerization of Hsp40s may lead to the recruitment of multiple Hsp70 molecules whose client binding-and-release cycles subsequently generate pulling forces that drive the dissolution of aggregates. For a detailed discussion of this model and alternative models, we refer the interested reader to [68,69].

#### 3.1.2. Insights from In Vivo Models of PMDs

An interesting question with respect to the tri-chaperone system is how increasing or decreasing its activity affects disease progression in vivo. It is known that that PMDs negatively affect overall proteostasis, which leads to secondary defects and toxicity [70]. Thus, boosting proteostasis capacity by upregulating components of the tri-chaperone system seems promising. However, if the tri-chaperone system mediates amyloid fragmentation as we and others propose, such upregulation might also boost the conversion of native protein and aggregate formation. In this scenario, beneficial effects due to increased proteostasis capacity would be accompanied by negative effects due to increased seed generation. Whether upregulation of the tri-chaperone system is protective in PMDs in vivo depends on the relative contribution of each of the mechanisms mentioned above. Below, we attempt to summarize previous studies in which key components of the tri-chaperone system have been modulated in in vivo models of PMDs.

Several studies have examined the loss of Hsp70 or Hsp110 in the context of PMDs [71,72,73]. Wacker et al. (2009) [71] used the R6/2 mouse model of Huntington’s disease and ablated the Hsp70 family members HSPA1A and HSPA2. Compared with R6/2-Hsp70^+/+^ mice, R6/2-Hsp70^−/−^ mice showed reduced survival, stronger motor deficits, and aggravated physical phenotypes (e.g., loss of body weight, worsened coat appearance). Interestingly, the size of inclusion bodies was increased in R6/2-Hsp70^−/−^ mice, but the size of fibrillar aggregates was unchanged. The authors also inoculated Hsp70^−/−^ mice with RML or 22L mouse-adapted scrapie prions, but interestingly, no effect on survival was observed. This contrasts with a recent study by Mays et al. (2019) [73], who reported a reduced incubation period and reduced median survival in *Hspa1a^−/−^*/*Hspa1b^−/−^* mice inoculated with RML prions. The differences between these two studies may have arisen from the different combinations of Hsp70 family members knocked out.

Other studies have examined the effects of Hsp70 or Hsp110 overexpression in the context of PMDs [74,75,76,77]. Most notably, Horwich and colleagues developed a mouse line that overexpresses the human Hsp110 family member Apg-1 (*HSPA4L*), which is hereafter referred to as TgApg-1 [76]. This line was subsequently crossed with a mouse line expressing mutated G85R SOD1 associated with amyotrophic lateral sclerosis (ALS) [76], and with a mouse line expressing PD-associated A53T α-syn [77]. In TgApg-1/G85R-SOD1 mice, the median survival was lengthened by approximately 2 months compared to littermate G85R-SOD1 controls. Similarly, TgApg-1/A53T-α-syn mice showed a 50-day extension of median survival compared to A53T-α-syn littermate controls. At 6 months of age, when A53T-α-syn mice normally develop motor symptoms and α-syn pathology, TgApg-1/A53T-α-syn mice also showed less α-syn accumulation in hippocampal CA1 neurons. Interestingly, the levels of oligomeric α-syn were not changed in TgApg-1/A53T-α-syn mice. While surprising given the in vitro data presented above, this finding does not exclude a role of Hsp110 in the fragmentation of α-syn aggregates in vivo. Instead, it is conceivable that enhanced proteostasis capacity due to Apg-1 overexpression facilitates the clearance of smaller aggregates or misfolded monomeric α-syn through alternative pathways such as autophagy or the ubiquitin–proteasome system. This appears especially likely, since the authors also report an unexpected increase in the expression of various Hsp70 and Hsp40 family members in TgApg-1 mice. Our own studies in TgApg-1 mice revealed a strain-specific protective role of Hsp110 overexpression in prion disease (Marrero-Winkens, C. and Schätzl H.M, personal communication). Specifically, we observed an approximately 6-day prolongation of mean survival in TgApg-1 mice inoculated with Me7 scrapie prions when compared to Me7-inoculated wild-type littermates. In contrast, 22L-inoculated TgApg-1 mice showed a small, non-significant prolongation of survival compared to 22L-inoculated WT mice. Similarly, Prusiner and colleagues observed a prolongation of survival in Hsp70 overexpressing mice inoculated with RML prions, but this was not statistically significant [75].

While some of the studies above found no effects upon the manipulation of Hsp70 or Hsp110, an overall picture emerges in which these chaperones are mostly protective in vivo, rather than being accelerators of disease. Given the complexities of in vivo models, it is difficult to determine whether Hsp70 or Hsp110 simply do not play a role in the propagation of amyloidogenic proteins in vivo, or whether such a function is merely overshadowed by broader beneficial effects on proteostasis. Mays et al. (2019) [73] have attempted to dissect these two distinct possible effects by examining prion conversion in addition to disease progression. Specifically, the authors used brain homogenates of uninfected *Hspa1a^−/−^*/*Hspa1b^−/−^* mice as a substrate for PMCA seeded with RML prions. This revealed that PrP^C^-to-PrP^Sc^ conversion is less efficient in Hsp70^−/−^ than WT brain homogenate. A possible interpretation of these results is that Hsp70 is involved in prion propagation, despite having an overall protective function in prion disease.

Further studies in this direction, combined with additional in vitro studies, as well as in vivo studies examining the kinetics of amyloid formation in genetically modulated animals, will reveal to which extent the tri-chaperone system participates in the propagation of amyloidogenic aggregates in vivo and how this relates to their overall function in proteostasis.

### 3.2. Macro-Autophagy

Macro-autophagy (hereafter referred to as autophagy) is a cellular process for the bulk degradation and recycling of cellular organelles and cytoplasmic proteins [78]. It is of particular importance during starvation, as the metabolites it provides can be used for biosynthetic processes or energy metabolism. Autophagy occurs via the sequestration of cytoplasmic substrates into developing double-membrane bound structures called autophagosomes. These subsequently fuse with late endosomes and lysosomes to form autolysosomes, in which the formerly cytosolic substrates are degraded. From late endosomes, there are two alternative pathways. One is the formation of membrane-bound extracellular vesicles known as exosomes via the inward budding of late endosomes/multivesicular bodies (MVBs). When MVBs fuse with the cell surface, exosomes are released and can deliver their contents to distant cells [79]. The second one is rab9-dependent recycling back to the trans-Golgi network (TGN), which ultimately delivers cargo to the secretory pathway [80]. Autophagy has been implicated in a range of diseases, both as a protective mechanism and as a contributing factor [81,82].

As we have recently discussed elsewhere, the induction of autophagy is generally protective in the context of prion diseases [83]. Our own work, and that of others, has shown that PrP^Sc^ is cleared from cultured cells following the induction of autophagy [84,85,86,87,88,89]. Similarly, autophagy inducers delay the appearance of PrP^Sc^ or extend the incubation period in prion-inoculated mice [87,90,91,92]. Studies in other PMDs have also found stimulating autophagy to be protective [93,94,95]. For instance, the induction of autophagy by rapamycin has been reported to reduce the levels of Aβ in Chinese hamster ovary (CHO) cells expressing mutant amyloid precursor protein (APP) [96] and to reduce the accumulation of Aβ as well as cognitive deficits in multiple distinct mouse models of AD [96,97]. Rapamycin was also found to reduce the levels of insoluble tau in COS-7 cells transfected with tau carrying the P301L mutation associated with FTD [98]. Similarly, studies in mouse models of tauopathies found autophagy inducers to reduce neuronal death, tau pathology, and behavioral deficits [99,100]. An enhanced clearance of α-syn following the induction of autophagy has also been reported for cultured cells expressing wild-type or mutant α-syn [101,102]. However, it should be noted that others have found an autophagy pathway distinct from macroautophagy, which is known as chaperone-mediated autophagy, to be of greater importance in the clearance of α-syn [93]. Nonetheless, a genetic or pharmacological induction of autophagy was protective in distinct mouse models of PD [103,104].

Overall, the evidence outlined above suggests that enhanced autophagy acts as a clearance mechanism rather than a ‘seed-generating’ fragmentation mechanism. However, during our own work on autophagy, we found evidence for basal (i.e., non-induced) autophagy as a requirement for establishing prion-infection de novo [105,106]. In these studies, we exposed prion-susceptible and non-susceptible cultured cells to brain homogenates from non-infected or prion-infected mice. Cells were lysed at fixed intervals post exposure, and the levels of PrP^Sc^ and LC3-II (a commonly used marker of autophagic flux) were determined. Susceptible cells in which de novo PrP^Sc^ was detected also showed a transient increase in LC3-II. This was not the case for susceptible cells exposed to uninfected brain or for non-susceptible cells exposed to either infected or uninfected brain. This finding suggests that de novo prion infection is accompanied by a brief induction in autophagy. To determine whether this transient increase in autophagy is required for de novo prion infection, we next exposed wild-type mouse embryonic fibroblasts (WT-MEF) and MEFs deficient in the autophagy regulator Atg5 (Atg5^−/−^-MEF) to prion-infected brain homogenate. To our surprise, only low levels of PrP^Sc^ were detected in Atg5^−/−^-MEFs at day 20, 30, and 50 post infection, while WT-MEF efficiently propagated PrP^Sc^ by this time. Re-introduction of Atg5 by the lentiviral transduction of Atg5^−/−^-MEF restored susceptibility to prion infection, underlining that this was not a clonal effect. Next, we extended our studies to neuronal and other non-neuronal cells. Autophagy was compromised in naïve cells by lentiviral delivery of siRNA against beclin-1 and Atg5 or by CRISPR–Cas9 mediated knockout of Atg-5. Subsequent prion infection was not affected in such autophagy-deficient cells. Combined with our data from Atg5^−/−^-MEF, this suggests that basal autophagy is required for establishing de novo prion infection in some cell types, but not others. It remains unclear how basal autophagy might promote de novo prion infection. One possibility is that basal autophagy causes fragmentation of PrP^Sc^ aggregates in late endosomes into new seeds, which are then partially recycled via the TGN to an upstream site of prion conversion, as has been shown by us and others [107,108,109,110].

Based on this work, we believe that the level of autophagic flux may determine how autophagy affects prion-like seeding of misfolded proteins. It is conceivable that basal autophagy only partially degrades fibrils—thus providing more seeding-competent species—while higher levels of autophagy, such as those observed after induction by lithium or rapamycin, favor the complete degradation of aggregates. While such a model would be consistent with our own experimental work on autophagy and that of others, it remains to be thoroughly validated by future research.

To the best of our knowledge, it has not yet been examined whether autophagy affects prion-like seeding of other proteins associated with PMDs, even though cell-to-cell and region-to-region spread are likely to occur via prion-like seeding [8]. One complication in studying this is the interrelation between autophagy and exosomal release [111]. We have shown for PrP^Sc^ that an induction of autophagy in stably infected cells results in a reduced exosomal release of PrP^Sc^ [112]. Similar observations had previously been made for the cell-to-cell transfer of α-syn in cultured cells [113,114]. Since changes in autophagy affect the clearance of aggregates on the one hand, and exosomal cell-to-cell spread on the other hand, manipulations in cells that are already burdened with aggregates will most likely not reveal whether autophagy provides an additional fragmentation function as we hypothesize. To examine this, specifically, it will be necessary to modulate autophagy in naïve recipient cells prior to the addition of exogenous propagation-competent aggregates. Such studies would also be enhanced by the inclusion of read-outs, which allow for a determination of aggregate size, e.g., sedimentation velocity-gradient centrifugation or field-flow fractionation [115,116]. We believe this to be a worthwhile endeavor, since it addresses the region-to-region spread that plays a major role in sporadic PMDs.

### 3.3. Proteasome–Autophagy Cross-Talk and Small Heat Shock Proteins (sHsps) as Indirect Contributors

Several other pathways may indirectly affect fragmentation by modulating autophagy or the Hsp70–Hsp40–Hsp110 system. Below, we will briefly discuss how UPS–autophagy crosstalk and sHsps may affect the propagation of amyloidogenic aggregates.

The UPS is a cellular mechanism for the targeted proteolytic degradation of ubiquitinated misfolded proteins [117]. Since it degrades soluble monomeric or low-*n* oligomeric substrates, a direct involvement in fragmentation is difficult to envision. An impairment of the UPS in various PMDs, including prion disease, AD, PD, and ALS [118,119], has been reported. For instance, Tabrizi and colleagues have shown that oligomeric PrP^Sc^ physically interacts with the 20S proteasome to inhibit gate opening and thus proper UPS function [120,121]. Similar mechanisms have been described for tau, Aβ, and α-syn [122,123,124]. Recent research has suggested that there may be ‘cross-talk’ between the UPS and autophagy, resulting in a compensatory upregulation of autophagy following UPS inhibition [125,126,127,128]. However, the precise nature of any UPS–autophagy cross-talk in PMDs and its overall contribution to protein aggregation remain to be determined in future research.

In mammals, sHsps are a family of 10 chaperones ranging in size from 12 to 43 kDa and are characterized by a common α-crystallin domain (ACD) with variable N- and C-terminal regions [129]. sHsps prevent the formation of large aggregates by interacting with intermediately folded proteins through hydrophobic residues. In addition to keeping substrates in near-native states, this reduces aggregate sizes. Combined, these effects of sHsp binding facilitate subsequent disaggregation and re-folding by other chaperones, including the mammalian Hsp70–Hsp40–Hsp110 system [59,67,130]. This suggests that similar to the Hsp70–Hsp40–Hsp110 system itself, sHsps may promote aggregate clearance on the one hand or seed generation on the other hand. Limited studies conducted in animal models of PMDs have generally found the overexpression of sHsps to be protective and loss to be detrimental [129]. However, this does not preclude a dual role at basal levels, especially considering that the role of both sHsps and the Hsp70–Hsp40–Hsp110 may shift throughout the time course of disease.

## 4. Conclusions

Fragmentation is an essential step in the propagation of prions and prion-like aggregates. This was suggested initially by kinetic models of prion conversion, and it is strongly supported by yeast prion biology and in vitro prion conversion assays. How fragmentation occurs in mammalian cells remains largely unknown. Here, we show that two mechanisms of aggregate clearance—namely the mammalian Hsp70–Hsp40–Hsp110 system and macro-autophagy—may not only be protective, but also play a role in aggregate fragmentation.

With regard to the mammalian Hsp70–Hsp40–Hsp110 system, recent work has shown that it can mediate the resolution of both amorphous aggregates and amyloids. Crucially, the tri-chaperone system was also found to generate not only monomeric species but also oligomeric seeding-competent aggregates in vitro. Further research showed that it affects seed generation and cell-to-cell spread in *C. elegans*, thus putting it on the map as a potential player in fragmentation. This appears to contrast with its largely protective role in vivo. However, such protection may be unrelated to its effects on pathogenic proteins; instead, it may be related to its overall benefit for proteostasis. We believe further in vitro studies as well as time-point experiments on aggregate formation in vivo will be beneficial to our understanding of the precise contribution of the Hsp70–Hsp40–Hsp110 system in prion diseases and prion-like protein misfolding diseases.

For autophagy, we present evidence from our own work suggesting that it may be required to establish de novo prion infection in some cell types. At first glance, this may seem to contrast with other work reviewed herein showing that autophagy clears aggregates. We propose that autophagy actually plays a dual role, and that its role may shift between seeding and aggregate clearance, which is possibly dependent on the level of autophagic flux. We suggest investigating autophagy modulation at different time points as an experimental avenue that may resolve the discrepancies presented here.

Overall, filling the knowledge gap that exists around fragmentation will be crucial to our understanding of human protein misfolding disorders, since most feature cell-to-cell and region-to-region spread via seeds generated from larger aggregates. Based on the evidence reviewed herein, we believe that the Hsp70–Hsp40–Hsp110 system and macro-autophagy represent promising avenues of research.

## Figures and Tables

**Table 1:** Selected protein misfolding diseases and the proteins involved.

Disease	Proteins
Alzheimer’s disease (AD)	β-amyloid (Aβ), tau
Pick’s disease (PiD)	Tau
Parkinson’s disease (PD)	α-synuclein (α-syn)
Lewy body dementia (LBD)	α-synuclein (α-syn)
Amyotrophic lateral sclerosis (ALS)	Superoxide dismutase 1 (SOD1), TAR DNA binding protein 43 (TDP-43), fused in sarcoma (FUS)
Polyglutamine diseases (PolyQ)	Various proteins, such as huntingtin
Prion diseases	Prion protein (PrP)

## Glossary

β-Amyloid (Aβ) and Alzheimer’s Disease (AD)

Aβ is a main driver behind AD pathogenesis and has been identified as the core component of extracellular plaques found in the brains of AD patients [131]. It derives from the larger amyloid precursor protein (APP) and is approximately 4–5 kDa in size. It is primarily found in tetramers and dimers, and oligomeric forms similar to these are now believed to be the primary source of neurotoxicity [132,133,134].

Tau, Alzheimer’s Disease (AD), and Pick’s Disease (PiD)

*MAPT* is a single gene with 16 exons encoding for the protein tau [135]. This protein is found in the core of intracellular neurofibrillary tangles (NFTs), which are commonly associated with AD and PiD [136]. Normally, tau is a soluble, phosphorylated protein, that is involved in axonal transport [137]. However, the hyper-phosphorylation of tau results in the formation of an insoluble, filamentous protein that aggregates in disease [138]. It has been shown that tau pathology gradually spreads throughout the brain over time, and the trans-synaptic transport of oligomeric tau ‘seeds’ has been linked to the spread of pathology [139]. Lastly, tau is associated with other diseases, collectively known as tauopathies, such as PiD—a frontotemporal dementia due to mutations in the *MAPT* gene [140].

α-Synuclein (α-syn), Parkinson’s Disease (PD), and Lewy Body Dementia (LBD)

α-syn is a 140 amino acid (aa) neuronal protein involved in neurotransmitter release and neuronal plasticity at the pre-synaptic terminal [141]. α-syn is encoded by the *SNCA* gene [142], which harbors mutations in some familial cases of Parkinson’s disease [142]. It is also the main component of Lewy bodies (LB), which are a common neuropathological feature of synucleinopathies such as PD and LBD [143].

Amyotrophic Lateral Sclerosis (ALS)

ALS is a neuromuscular degenerative disorder, involving the formation of protein aggregates [144]. This disease has a strong genetic component, and most forms of familial ALS (fALS) arise from genes involved in RNA metabolism, cytoskeletal dynamics, and proteostasis [145]. The most common genes are TAR DNA binding protein 43 (*TDP-43*), fused in sarcoma (*FUS*), and superoxide dismutase 1 (*SOD1*) [146,147,148,149]. Of these, the most prion-like protein is SOD1, a Cu/Zn binding superoxide dismutase of 32 kDa that catalyzes the dismutation of reactive oxygen species to non-radical species [144]. Many gain-of-function mutations in *SOD1* result in fALS. Such mutations have been shown to cause misfolding, aggregation, fibril formation, seeding, cell-to-cell propagation, and even animal-to-animal propagation [149,150,151,152,153].

The Prion Protein (PrP) and Prion Diseases

Prion diseases, also known as transmissible spongiform encephalopathies (TSEs), are a group of invariably fatal neurodegenerative diseases in humans and animals. Examples include bovine spongiform encephalopathy (BSE) in cattle, scrapie in sheep, chronic wasting disease (CWD) in cervids, and Creutzfeldt–Jakob disease (CJD) in humans [154,155,156,157]. Prion diseases are caused by the misfolding of the cellular prion protein (PrP^C^) into its pathogenic isoform (PrP^Sc^), which is the sole constituent of the prion agent causing TSEs [4]. PrP^Sc^ is highly resistant to inactivation, and it gives rise to aggregates as well as amyloidogenic fibrils [4,158]. The highly infectious character of prions at the cellular and organismal level sets prion disorders apart from other PMDs.
